# Interactions Between Rumen Microbes, VFAs, and Host Genes Regulate Nutrient Absorption and Epithelial Barrier Function During Cold Season Nutritional Stress in Tibetan Sheep

**DOI:** 10.3389/fmicb.2020.593062

**Published:** 2020-11-05

**Authors:** Xiu Liu, Yuzhu Sha, Renqing Dingkao, Wei Zhang, Weibing Lv, Hong Wei, Hao Shi, Jiang Hu, Jiqing Wang, Shaobin Li, Zhiyun Hao, Yuzhu Luo

**Affiliations:** ^1^College of Animal Science and Technology, Gansu Key Laboratory of Herbivorous Animal Biotechnology, Gansu Agricultural University, Lanzhou, China; ^2^Gannan Institute of Animal Husbandry Science, Hezuo, China

**Keywords:** cold season, nutritional stress, rumen microorganisms, volatile fatty acids, Tibetan sheep

## Abstract

As one of the important ruminants of the Qinghai-Tibet Plateau, Tibetan sheep are able to reproduce and maintain their population in this harsh environment of extreme cold and low oxygen. However, the adaptive mechanism of Tibetan sheep when nutrients are scarce in the cold season of the Plateau environment is unclear. We conducted comparative analysis rumen fermentation parameters, rumen microbes, and expression of host genes related to nutrient absorption and rumen epithelial barrier function in cold and warm season Tibetan sheep. We found that concentrations of the volatile fatty acids (VFAs) acetate, propionate and butyrate of Tibetan sheep in the cold season were significantly higher than in the warm season (*P* < 0.05). Microbial 16S rRNA gene analysis revealed significant differences in rumen microbiota between the cold and warm seasons, and the abundance of microbial in the cold season was significantly higher than that in the warm season (*P* < 0.05), and the lack of nutrients in the cold season led to a significant reduction in the expression of *SGLT1*, *Claudin-4*, and *ZO-1* genes in the rumen epithelium. Correlation analysis revealed significant associations of some rumen microorganisms with the fermentation product acetate and the rumen epithelial genes *SGLT1*, *Claudin-4*, and *ZO-1*.

## Introduction

Microbiotas are symbiotic with all macroorganisms. The growth, development, and health of macroorganisms are affected by microbes in their environment that determine the physiology, behavior, ecology, and evolution of the host (Hadfield et al., 2013; Foster et al., 2017). Biology is undergoing a paradigm shift, and individual phenotypes are now regarded as the result of interactions between the host genome and related microbiome. The mammalian digestive system is colonized by complex microorganisms, including bacteria, archaea, fungi, and protozoa, which play an important role in diet fermentation and host energy supply (Lin et al., 2019). There is a strong symbiotic relationship between animal gut microbiota and the host, which helps the host digest and assimilate food, and thereby provides energy and nutrition to the host. For example, intestinal microorganisms can aid the decomposition of cellulose, hemicellulose, and other non-digestible substances in the host, so that these compounds can be utilized by the host (Hooper et al., 2002). The rumen epithelium is a unique location for host-microorganism interactions, and these interactions can affect the net use of nutrients by the host (Holmes et al., 2012; Malmuthuge and Guan, 2017). The availability of food, nutrient intake patterns of animals, and geographical resources change with time and season. Different food sources or geographical environments have significant effects on microbial composition in the host gut (Amato et al., 2015; Sun et al., 2016).

Tibetan sheep, bred on the Qinghai-Tibet Plateau at an altitude of more than 3,000 m, are the main source of meat, wool, fuel, and other necessities for the economy and lifestyle of the local population. Tibetan sheep are in a state of natural grazing all year round, and they consume natural herbage as their predominant source of nutrients (Wiener et al., 2011; An et al., 2005). Alpine grassland of the Qinghai-Tibet Plateau experiences two seasons, warm and cold, which correspond to changes in the grassland vegetation in the grass and hay periods. The duration of the grass period (warm season) is less than 5 months; the grass starts to turn green in early May and turns yellow in early October. Throughout the year, Tibetan sheep mainly acquire nutrients by eating natural herbage, but during the long hay period (cold season); they can only acquire nutrients by eating dry grass. Despite these harsh environmental conditions, Tibetan sheep survive and maintain their population. However, how Tibetan sheep adapt to the harsh Plateau environment is unclear and could be due to various factors, the most important of which may be genetic factors. Most previous studies on plateau adaptation have focused on host genomes, because genetic adaptation is the central focus of evolutionary biology, and only a few studies have been conducted on microbiota and its coevolution (Zhang et al., 2016). However, a growing number of studies have found that microbial cells inhabiting the host gastrointestinal tract play an important role in host adaptation (Kwong et al., 2017; Ross et al., 2018; Zepeda Mendoza et al., 2018). Lin et al. (2019) emphasized the importance of the joint development of the host and its microbiome on the adaptability of the host, and the identification of a common set of genes that regulate host-microorganism interactions between mammalian populations and species have broad implications for human health and animal biology (Lin et al., 2019; Suzuki et al., 2019). Therefore, combined with the study of microbiome, it can better explain the plateau adaptability of Tibetan sheep in cold season.

Mammalian gastrointestinal microorganisms also play an important role in epithelial barrier function, and provide many benefits to the host, including nutrient utilization and protection against pathogens via the secretion of compounds that kill or inhibit the undesirable organisms (Yoon et al., 2014). For example, carbohydrates are effectively decomposed into short-chain fatty acids by commensal microorganisms in the rumen for use by the host. However, the situation with glucose is unique amongst these carbohydrates. In the rumen, most glucose is decomposed into short-chain fatty acids by commensal microorganisms. However, the ion transporter SGLT-1 can directly transfer some glucose from the rumen to the blood for absorption, thereby saving energy (Aschenbach et al., 2000a, b, 2002). In addition, the rumen epithelial barrier comprises a network of intercellular protein complexes that form selective tight junctions (TJs) and can interact with the epithelial microbiome (Zhang et al., 2018). Claudins are key molecules in the barrier function of TJs. ZO-1, the first tight junction protein to be discovered, interacts with various other connecting components and plays a pivotal role (Furuse et al., 1998; Bazzoni et al., 2000; Zhang et al., 2018). This indicates that there is a distinct relationship between rumen microorganisms and epithelial barrier function and nutrient absorption in the host. However, there are limited studies on the changes of rumen microorganisms in Tibetan sheep grazing on the Qinghai-Tibet Plateau in the cold and warm seasons, and the expression of genes related to nutrient absorption and epithelial barrier function in the rumen of these sheep. At present, most of the studies only focus on the diversity of rumen microorganisms, while few studies explain the plateau adaptability in cold season through the combination of microbiome and genome. The current study aimed to address this by obtaining the rumen fermentation profile and rumen microbiota of Tibetan sheep under cold season nutrient stress by analyzing changes in the rumen internal environment (rumen fermentation mode, 16S rRNA gene sequencing) in cold and warm seasons, and examining expression of related genes in rumen epithelial tissues. Changes in gene expression associated with the rumen epithelium may provide new insights into the interactions between rumen microbes-volatile fatty acids (VFAs)-host genes, and thus could help elucidate the cold season adaptability of Tibetan sheep.

## Materials and Methods

### Ethics Statement

All studies involving animal were carried out in accordance with the regulations for the Administration of Affairs Concerning Experimental Animal (Ministry of Science and Technology, China; revise in June 2004), and sample collection protocols were approved by the Livestock Care Committee of Gansu Agricultural University (Approval No. GAU-LC-2020-27).

### Experimental Design and Sampling

Ten healthy Tibetan sheep ewes (1 year old, weight = 35.12 ± 1.43 kg) were obtained from a farm in Gannan Tibetan Autonomous Prefecture (Gansu Province, China), which used local traditional natural grazing management and was located at an altitude of 3,300 m. These animals are randomly divided into two groups, representing the warm (July) and cold (December) seasons, with five sheep in each period, grazing in the same pasture as the other sheep. Samples were collected in July 2019 and December 2019, respectively. Rumen samples (50 ml, rumen fluid) were collected from each animal with a gastric tube rumen sampler in the morning and were immediately frozen in liquid nitrogen and stored at −80°C for 16S rRNA sequencing. Then the jugular vein bloodletting was performed, and the internal organs and digestive tract were carefully dissected after death, the rumen contents were taken immediately (for determination of VFAs), and rumen epithelial tissue samples were acquired by dissecting a small piece of rumen abdominal sac, quickly removing rumen contents by rinsing with phosphate-buffered saline (PBS), then separating the epithelial tissue with blunt scissors. Tissue samples were placed in liquid nitrogen and stored at −80°C refrigerator for subsequent RNA extraction.

### Measurement of Volatile Fatty Acids

The collected rumen fluid was centrifuged at 5400 rpm for 10 min, 1 ml of supernatant was pipetted into a 1.5 ml centrifuge tube, and then 0.2 ml of 25% metaphosphoric acid solution containing internal standard 2EB was added. Then mix, ice bath for 30 min, centrifuge at 10000 rpm for 10 min, filter the supernatant into a sample bottle with a 0.25 um filter, and wait for the gas chromatograph to determine (GC-2010 plus; Shimadzu, Japan). The internal standard method was adopted, using 2-ethyl butyric acid (2EB) as the internal standard. The chromatographic column was an AT-FFAP (50 m × 0.32 mm × 0.25 m) capillary column. The column temperature was maintained at 60°C for 1 min, then increased to 115°C at 5°C/min without reservation, and then increased to 180°C at 15°C/min. The detector temperature was 260°C and the injector temperature was 250°C.

### DNA Extraction and High-Throughput Sequencing

Total DNA was isolated from each rumen sample using MN NucleoSpin 96 Soil kit (Macherey-Nagel, Germany). First, appropriate rumen contents were transferred to a 2 mL centrifuge tube and lysate was added to make the samples fully decomposed. The samples were centrifuged at 1200 rpm for 2 min and the supernatant was transferred. Then, extraction was carried out according to the specific steps of the kit. The bacterial V3–V4 region of 16S rRNA genes in the total DNA were amplified using the primers 338F (5′-ACTCCTACGGGAGGCAGCAG-3′) and 806R (5′-GGACTACHVGGGTWTCTAAT-3′). The PCR conditions comprised a pre-denaturation step at 95°C for 3 min, 40 cycles of denaturation at 95°C for 30 s, annealing at 55°C for 30 s, and extension at 72°C for 30 s, followed by a final extension step at 72°C for 7 min. All amplified products were sequenced and analyzed on an Illumina MiSeq platform (Illumina, San Diego, CA, United States). The sequencing data were deposited into the Sequence Read Archive (SRA) of NCBI (Accession No. SRR12719079-SRR12719088).

### RNA Extraction and Gene Expression Analysis of Rumen Epithelium

Total RNA was extracted from rumen epithelial tissues of Tibetan sheep using the Trizol reagent method (TransGen). cDNA synthesis was performed using a reverse transcription kit containing the gDNA wiper enzyme. Primer 5.0 software was used to design primers for the genes *SGLT1*, *Claudin-4* and *ZO-1*; β-actin was the internal reference gene. Primer information is shown in Table S1. An Applied Biosystems Q6 Quantitative PCR instrument was used to quantify fluorescence of the rumen epithelium-related genes and the internal reference gene. Reaction conditions were pre-denaturation at 95°C for 30 s, 40 cycles of 95°C for 10 s and 60°C for 30 s, and the dissolution curve (95°C for 15 s, 60°C for 60 s, 95°C for 15 s). The 20-μl reaction system contained 2 × ChamQ Universal SYBR qPCR Master Mix, cDNA template and upstream and downstream primers. The resulting data were analyzed using the 2^–ΔΔ*CT*^ method and β-actin as the internal reference gene for correction (Livak and Schmittgen, 2001).

### Bioinformatics Analysis

Based on the raw data returned by the Illumina HiSeq sequencing platform, double-ended splicing (FLASH v1.2.7), filtering (Trimmomatic v0.33), and removal of chimeras (UCHIME v4.2) were used to obtain optimized sequences (tags). Usearch software (Edgar, 2013) was utilized to cluster tags at a similarity level of 97%, obtain operational taxonomic units (OTUs), and annotate the OTUs based on the Silva (bacterial) taxonomy database. Based on the results of OTU analysis, taxonomic analysis was performed on the samples at various classification levels to obtain community structure maps, species clustering heat maps, and taxonomy of each sample at the level of phylum, class, order, family, genus, and species. Alpha diversity was used to analyze species diversity within a single sample, the diversity index Ace, Chao1 (the measurement of species abundance), and Shannon, Simpson (the measurement of species diversity) were obtained, and the samples were plotted as a dilution curve (Wang et al., 2012) and rank abundance curve. Beta diversity analysis was used to compare differences in species diversity (microbial composition and structure) between different samples. Obtain the sample level clustering (UPGMA) tree, NMDS analysis, sample clustering heat map and sample PCA, PCoA map (Sakaki et al., 1994; Dubois et al., 2010) (with grouping information), box plot based on multiple distances, etc. according to the distance matrix; Through the significance analysis of the difference between groups (LEfSe analysis), look for Biomarker with statistical differences between different groups (Segata et al., 2011), and use Metastats software to perform a *T* test on the species abundance data between the groups (White et al., 2009); get *p* value, pass Correct the *p* value to obtain the *q* value; finally select the species that lead to the difference in the composition of the two groups of samples according to the *p* value or the *q* value; through 16S functional gene prediction analysis (KEGG and COG), predict the gene function of the sample and calculate the functional gene abundance.

### Statistical Data Analysis

The independent sample *T* test in SPSS software (version 24.0, SPSS Inc.) was used to analyze differences in rumen fermentation parameters (VFAs) and related gene expression in the cold and warm seasons; *P* < 0.05 indicated a significant difference. The Spearman correlation test was used to analyze the correlation between VFAs, gene expression, and rumen microorganisms (relative abundance >0.5%).

## Results

### Determination of Rumen Fermentation Parameters

Various rumen fermentation parameters of Tibetan sheep were measured in different seasons (Table 1). The concentration of some VFAs were significantly higher in the cold season (December) than in the warm season (July) (*P* < 0.05). These VFAs included acetate, propionate, and butyrate; other VFAs had no significant difference in concentrations between the cold and warm seasons (*P* = 0.240). Proportionate analysis of the VFAs revealed that there was no significant difference in the proportion of propionate between cold and warm seasons (*P* = 0.127). The proportion of acetate in the cold season was significantly higher than that in the warm season, while the proportions of butyrate and other VFAs were significantly higher in the warm season than the cold season (*P* < 0.05). The ratio of acetic acid to propionic acid (A:P) was significantly higher in the cold season than in the warm season (*P* < 0.05).

**TABLE 1:** Effects of cold season nutritional stress on rumen fermentation parameters.

Ruminal parameter	Cold season	Warm season	*P*
**Concentration (mmol/L)**			
Acetate	57.59 ± 5.89	33.62 ± 1.25	<0.001
Propionate	16.54 ± 1.42	12.80 ± 0.29	<0.001
Butyrate	7.76 ± 0.35	6.72 ± 0.23	0.001
Other VFAs	3.16 ± 0.10	3.22 ± 0.04	0.240
Total VFA	85.05 ± 7.63	56.36 ± 1.35	<0.001
**Proportion (%)**			
Acetate	56.32 ± 0.008	46.53 ± 0.013	<0.001
Propionate	19.27 ± 0.010	18.41 ± 0.006	0.127
Butyrate	17.55 ± 0.009	23.63 ± 0.020	<0.001
Other VFAs	6.86 ± 0.009	11.44 ± 0.000	<0.001
Acetate: Propionate	2.93 ± 0.107	2.53 ± 0.009	<0.001

*Other VFAs: the sum of Valerate, Isobutyrate, and Isovalerate. Values are the mean ± standard deviation.*

### Diversity of Rumen Microbes

**FIGURE 1:**
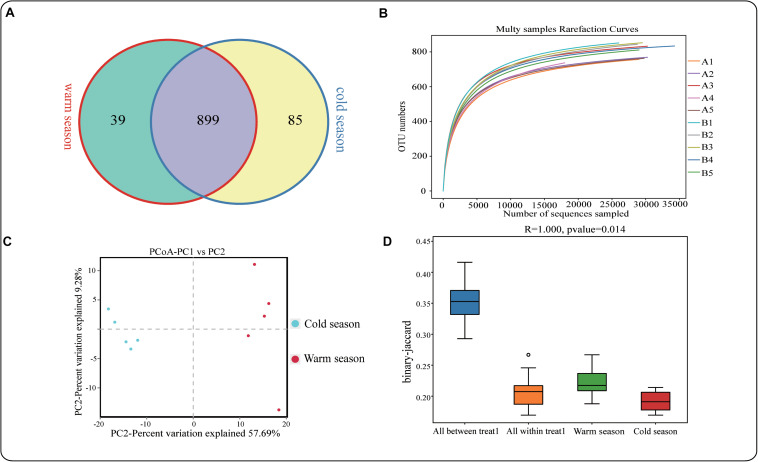
**(A)** OTU-Venn diagram analysis of cold and warm seasons; **(B)** Dilution curve analysis; **(C,D)** PCoA and Anosim analysis.

A total of 798,832 pairs of reads were obtained in this study, and 742,226 clean tags were generated after tiling and filtering of double-ended reads. At least 73,683 (average 74,223) clean tags were generated for each sample, with an average sequence length of 419 bp (Supplementary Table S2). Usearch software was used to cluster tags at the similarity level of 97%, OTUs for each sample was obtained. A total of 1023 OTU were obtained, including 938 in the warm season and 984 in the cold season (Figure 1A). The number of unique OTU in the cold season was significantly higher than that in the warm season. The dilution curve described the species diversity and species richness of each sample. The curve flattened at 20,000 reads, indicating that the sequencing coverage was saturated (Figure 1B). As shown in Table 2, the alpha-diversity index indicated that ACE in the cold season was significantly higher than that in warm season (*P* < 0.05), suggesting that microbial species abundance in the rumen of Tibetan sheep was significantly higher in the cold season than in the warm season. Shannon and Chao1 indexes were also higher in the cold season than in warm season, but the differences were not significant. The species accumulation curve (Figure S1) shows that the numbers of species and common species in the environment have reached saturation and will not increase further with any further increases of sample size. PCoA analysis revealed significant differences in rumen microbial species between the cold season and the warm season (Figure 1C). Anosim analysis further showed that the differences between groups were significantly greater than those within groups (Figure 1D).

**TABLE 2:** Effects of nutritional stress in cold season on diversity index.

Index	Warm season	Cold season	*P*
Shannon	5.2107	5.3060	0.4820
Simpson	0.0208	0.0161	0.5657
ACE	824.8360	882.5447	0.0063
Chao1	851.7650	900.9231	0.0646

### The Impact of Cold Season Nutritional Stress on Rumen Microbial Composition

**FIGURE 2:**
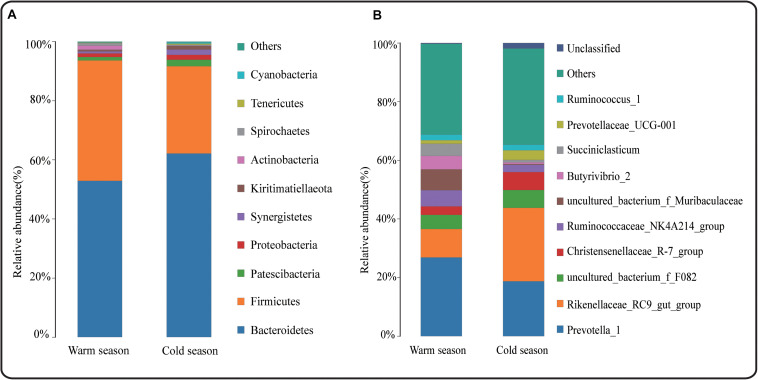
**(A)** Relative abundance of phylum horizontal species; **(B)** Relative abundance of genus horizontal species.

**FIGURE 3:**
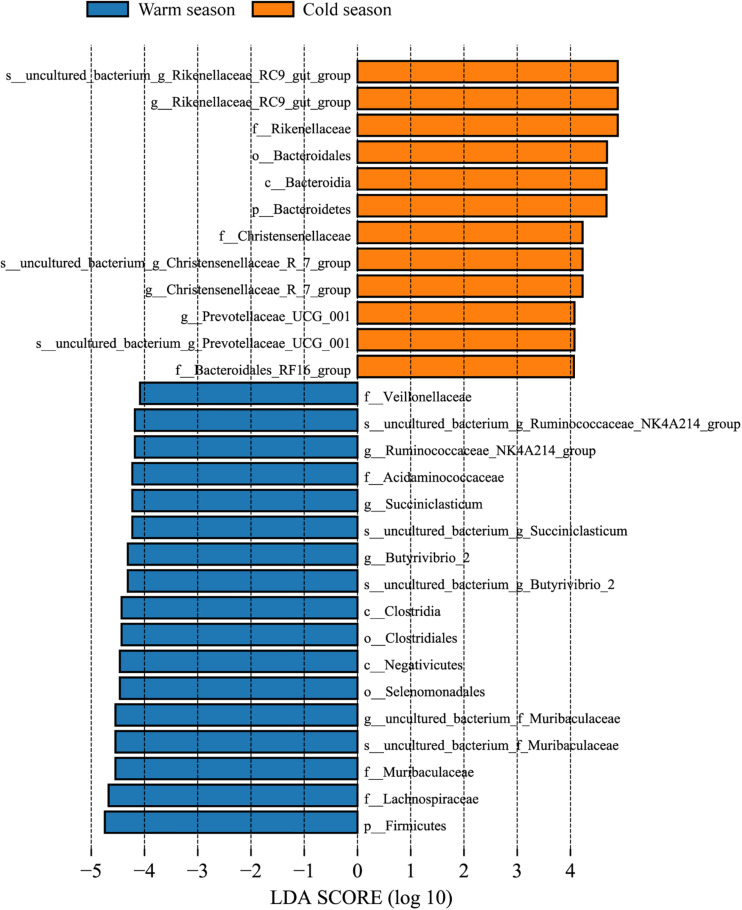
LDA value distribution histogram. LDA value > 4, and the length of the bar chart represents the influence of different species.

At the taxonomic level, a total of 17 phyla, 26 classes, 42 orders, 68 families, 160 genera, and 175 species were detected in the rumen microbiota. At the phylum level, Bacteroidetes, Firmicutes, Patescibacteria, Proteobacteria, and Actinobacteria were the dominant bacteria, and their relative abundances were all greater than 1%. Bacteroidetes and Firmicutes had the highest relative abundances in both cold and warm seasons, accounting for more than 90% of the total rumen bacteria (Figure 2A). In addition, there were 10 differential phyla between the cold and warm seasons (Supplementary Data Sheet 1), among which six phyla had a relative abundance that was significantly higher in the cold season than in the warm season (*q* < 0.05), and four phyla had a relative abundance that was significantly lower in the cold season than in the warm season. The most obvious of these differential phyla were Bacteroidetes, which increased significantly in the cold season (*q* = 0.0153), and Firmicutes, which decreased significantly in the cold season (*q* = 0.0063). At the genus level (Figure 2B), the relative abundance of bacteria in 66 genera was more than 0.1%, and *Prevotella_1* and *Rikenellaceae_RC9_gut_group* were the dominant genera in both cold and warm seasons. A total of 66 differential genera were identified among 160 genera (*q* < 0.05) (Supplementary Data Sheet 1). Of the 10 most abundant genera, *Rikenellaceae_RC9_gut_group* showed a significant increase in relative abundance in the cold season (*q* < 0.05), while the relative abundances of *Ruminococcaceae_NK4A214_group*, *uncultured_bacterium_f_Muribaculaceae*, *Butyrivibrio_2*, and *Succiniclasticum* were significantly reduced in the cold season (*q* < 0.05). Many cellulose-decomposing bacteria were also identified in this study, such as *Ruminococcus_2*, *Fibrobacter*, *Butyrivibrio_2*, *Treponema_2*, and *Pseudobutyrivibrio*. ANOVA analysis revealed significant differences in the rumen microbiota between cold and warm seasons at the phylum and genus levels (Supplementary Data Sheet 1). LEfSe analysis of samples between groups showed that there were 12 differential biomarkers (LDA score >4) for the two seasons (Figure 3), which was in agreement with the ANOVA analysis. Overall, there were significant differences between cold season and warm season.

### Gene Function Prediction

**FIGURE 4:**
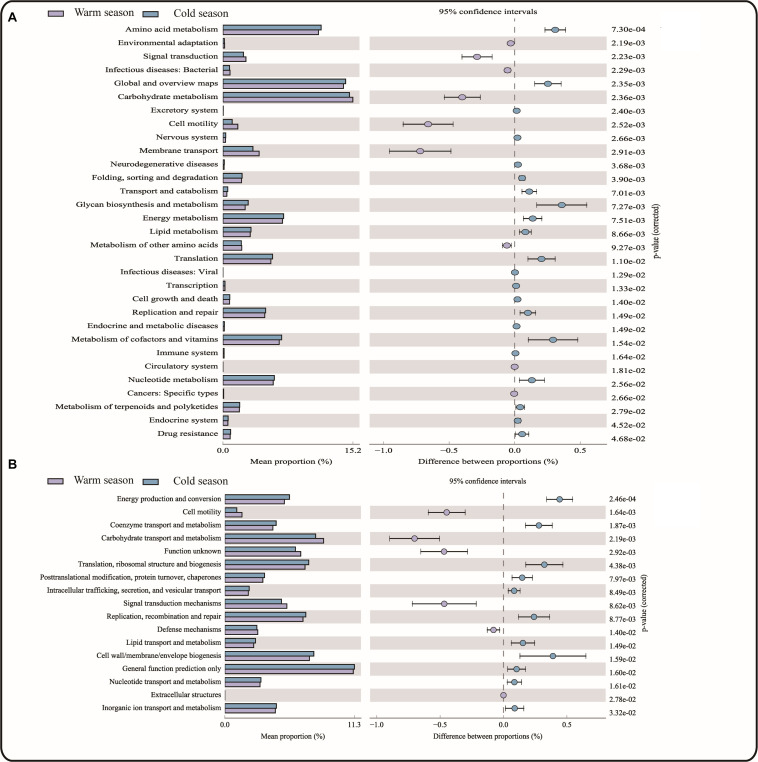
**(A)** KEGG functional pathway; **(B)** COG functional pathway.

A total of 43 KEGG gene families and 25 COG gene families were identified in the 16S rRNA gene sequencing data using PICRUSt software to predict gene function. Among these predictions, 31 KEGG gene families and 13 COG gene families showed significant differences between the cold and warm seasons (Figure 4). Of the 43 KEGG gene families, more than 70% were classified as metabolism-related, with functions related to carbohydrate metabolism accounting for the largest proportion (15.19% in warm season and 14.79% in cold season), followed by amino acid metabolism and energy metabolism. Energy metabolism-related functions were significantly increased in the cold season (*P* = 0.0075) compared with the warm season, and the corresponding glucose biosynthesis and metabolism-related functions were also significantly increased in the cold season (*P* = 0.0073). In addition, gene families involved in Immune system functions were significantly increased in the cold season compared with the warm season. However, the functions related to Membrane transport and Environmental adaptation were significantly reduced in the cold season. Among the 13 significantly different COG gene families, the functions related to Carbohydrate transport and metabolism were significantly decreased in the cold season (*P* = 0.0022), while Energy production and conversion was significantly increased in the cold season (*P* = 0.0002). This was consistent with the KEGG pathway analysis.

### mRNA Expression Levels in the Rumen Epithelium

**FIGURE 5:**
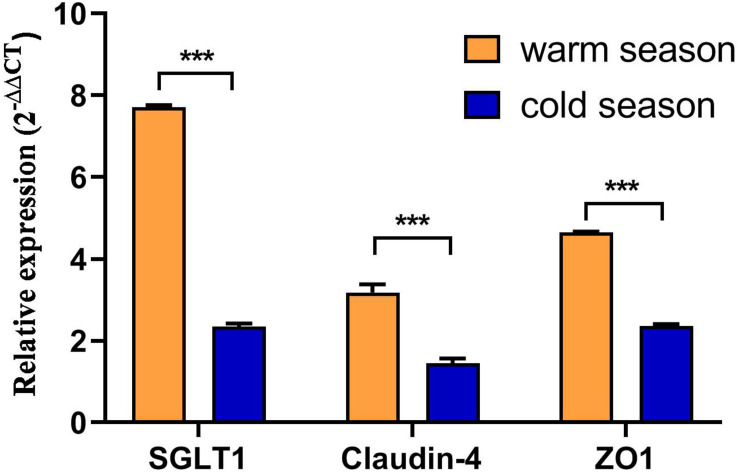
Nutrient absorption and barrier function related gene expression. ***P < 0.001.

As shown in Figure 5, expression of the nutrient absorption-related gene *SGLT1* in the rumen epithelium during the cold season was 3.2 times lower than that in the warm season and this was a significant difference (*P* < 0.001). However, expression of the genes related to the rumen epithelial barrier, *Claudin-4* and *ZO-1*, was significantly lower in the cold season than in the warm season (*P* < 0.001). Moreover, expression of *ZO-1* in the rumen epithelium was significantly higher than that of *Claudin-4* (*P* < 0.001).

### Interactions Between Expressions of Genes Related to Nutrient Absorption and Barrier Function in the Rumen Epithelium, VFAs, and Rumen Microorganisms

**FIGURE 6:**
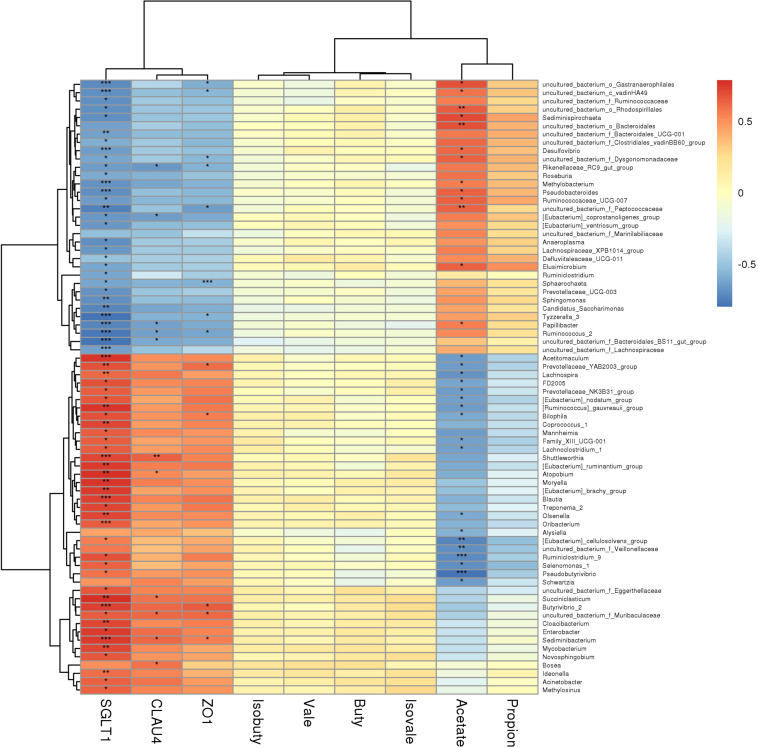
Correlation heat map. *P < 0.05, **P < 0.01, ***P < 0.001.

**FIGURE 7:**
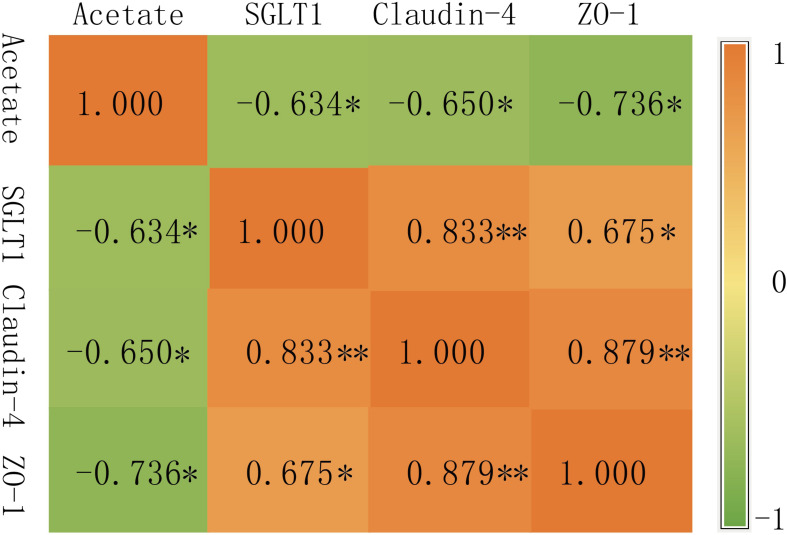
Correlation between genes. ^∗^ At the 0.05 level (two-tailed), the correlation is significant; ^∗∗^ At the 0.01 level (two-tailed), the correlation is significant.

The rumen horizontal microbial community data (relative abundance >0.5%) and the expression of rumen VFAs and rumen epithelial mRNA (2^–ΔΔ*CT*^) data sets were used to construct a heat map (correlation threshold >0.5) (Figure 6 and Figure S2); Figure 6 is the heat map of all microorganisms with a significant correlation of less than 0.05 (*P* < 0.05). Acetate was clustered with propionate, while the genes *SGLT1*, *Claudin-4*, and *ZO-1* were clustered together. In the rumen VFAs, only acetate was significantly correlated with genera-level microorganisms (*P* < 0.05). Of the 35 bacterial genera that were significantly correlated with acetate, 16 were positively correlated and 19 were negatively correlated. *Moraxella*, *Pseudobutyrivibrio*, and *Ruminiclostridium_9* were negatively correlated with acetate in a highly significant way (*P* < 0.001), and the correlation coefficients were all greater than 0.7. Among the three rumen epithelium-related genes, *SGLT1* was significantly correlated with 83 bacterial genera (*P* < 0.05); 46 were positively correlated with *SGLT1* and 37 were negatively correlated. *Claudin-4* and *ZO-1* were significantly correlated with 13 bacterial genera (*P* < 0.05). For *Claudin-4*, seven genera were positively correlated with expression of this gene and six were negatively correlated, while five genera were positively correlated with expression of *ZO-1* and eight genera were negatively correlated. In addition, 17 bacterial genera showed highly significant correlation with *SGLT1* expression (*P* < 0.001), but of the genera that were significantly correlated with expression of *Claudin-4* and/or *ZO-1*, only *Sphaerochaeta* showed highly significant correlation with *ZO-1* (*P* < 0.001). From the correlation heat map (Figure 6), it could be concluded that most of the microorganisms that were correlated with acetate showed the opposite correlation with expression of the three rumen epithelial genes. Therefore, the correlation between acetate and expression of *SGLT1*, *Claudin-4*, and *ZO-1* was further analyzed (Figure 7). There was significant negative correlation between acetate and the three genes (*P* < 0.05), with correlation coefficients greater than 0.6. In addition, there was significant positive correlation between *SGLT1*, *Claudin-4*, and *ZO-1* (*P* < 0.05), and *Claudin-4* displayed significant correlation with *SGLT1* and *ZO-1* at the level of *P* < 0.01, with correlation coefficients of 0.833 and 0.879, respectively.

## Discussion

Tibetan sheep are a ruminant of the Qinghai-Tibet Plateau and can survive in the harsh environment and maintain their population through reproduction, which has a certain relationship with the host genome (Wei et al., 2016). However, there is also an association with the microbiome, known as the “second genome” (Zhang et al., 2016), and this may be an important adaptive mechanism (Sun et al., 2016). Studies have reported that more than 75% of VFAs are absorbed by the rumen epithelium and form the main source of energy for ruminants (Baldwin, 1998; Russell and Rychlik, 2001). In this study, the total VFA in the cold season was significantly higher than that in the warm season (*P* < 0.05), which may be caused by Tibetan sheep showing high VFA in order to adapt to the nutrient deficiency in the cold season and provide energy source for the body. The concentrations of acetate, propionate and butyrate in rumen of Tibetan sheep in the cold season were significantly higher than those in the warm season (*P* < 0.05), the difference of other VFAs was not significant. Polyorach et al. (2014) found that high levels of nutrition and diet intake can reduce the concentration of acetate, significantly increase the proportion of propionate, which significantly reduces the A:P ratio (Polyorach et al., 2014). Because the warm season forage is more abundant than the cold season, the Tibetan sheep in this study eat a large amount of high-nutrient forage in the warm season, resulting in a significantly lower acetate content than the cold season, which is consistent with the results of Polyorach et al. (2014). However, the propionate concentration was significantly higher in the cold season than in the warm season, but the ratio of A:P was significantly lower in the warm season than in the cold season. From the perspective of energy utilization, the lower the A:P ratio, the higher the energy efficiency of the feed. In this study, the A:P ratio of grazing Tibetan sheep in both the cold and warm seasons was lower than that of drylot-feeding Tibetan sheep (C:F = 0:100 (concentrate to forage ratio), A:P = 6.38) (Liu et al., 2019), indicating that grazing Tibetan sheep are able to use the higher energy efficiency of their pastures. In summary, Tibetan sheep evolved a strong energy adaptation mechanism to cope with nutrient deficiency in the cold season. It has been reported that there is correlation between VFAs and rumen microorganisms (Lin et al., 2019). Sequencing the microorganisms in the rumen of grazing Tibetan sheep during the cold and warm seasons revealed that there was a significant difference in rumen microbial diversity between the two seasons, and that microbial species abundance in the cold season was significantly higher than that in the warm season (*P* < 0.05). Further analysis using PCoA and Anosim showed that there was a significant difference between the cold and warm seasons, and the difference between the seasonal groups was significantly greater than that within the groups (*P* < 0.05). This may be due to the lack of food in the cold season, leading to a large difference in the acquisition of food resources (Sun et al., 2016), as seen in previous studies (Zeng et al., 2017; Zhou et al., 2017; Xue et al., 2018; Hu et al., 2019).

In this study, Bacteroidetes and Firmicutes were dominant at the phylum level. Bacteroidetes increased significantly in the cold season (*q* = 0.0153), while Firmicutes were significantly reduced in the cold season (*q* = 0.0063). The main function of members of the phylum Bacteroides in the host is to degrade carbohydrates and proteins (Fernando et al., 2010; Jami et al., 2014; Nuriel-Ohayon et al., 2016). Firmicutes carry many genes encoding enzymes related to energy metabolism, which can produce many digestive enzymes to break down various substances, thereby helping the host to digest and absorb nutrients (Kaakoush, 2015). Consequently, a high ratio of F/B (Firmicutes/Bacteroides) can help the host effectively absorb energy-related substances and maintain the metabolic balance in low-temperature environments (Ley et al., 2006; Fernando et al., 2010; Murphy et al., 2010). In this study, the F/B ratio of the Tibetan sheep in the cold season was lower than that in the warm season, and this may be related to the lack of nutrients in the cold season, resulting in insufficient energy. However, the F/B value of grazing Tibetan sheep in the cold season (0.47) was higher than that of drylot-feeding Tibetan sheep (Liu et al., 2019) (C:F = 0:100, F/B = 0.27), indicating that grazing Tibetan sheep have adapted to the harsh environment of the cold season on the Plateau. Patescibacteria, Proteobacteria and Actinobacteria formed the second group of dominant bacterial phyla. Studies have shown that *Proteobacteria* is related to energy accumulation (Bryant and Small, 1956; Koren et al., 2012; Amato et al., 2014; Chevalier et al., 2015). The abundance of *Proteobacteria* was higher in the cold season than in the warm season, but the difference was not significant (*P* > 0.05). This trend was consistent with the results of Sun et al. (2016) who found that the abundance of *Proteobacteria* in winter was significantly higher than that in spring. Therefore, we infer that grazing Tibetan sheep require increased energy accumulation in order to adapt to the harsh environment of the cold season.

At the genus level, *Prevotella_1* and *Rikenellaceae_RC9_gut_group* were the dominant genera in the rumen of Tibetan sheep. The abundance of *Prevotella_1* was lower in the cold season than in the warm season, but the difference was not significant (*P* > 0.05). The abundance of *Prevotella_1* is related to changes in diet and nutrition; for example, a reduction in the amount of protein and starch can lead to a decrease in the abundance of *Prevotella_1* (Stevenson and Weimer, 2007). In addition, the genus *Prevotella_1* is considered to be related to the production of propionic acid (Strobel, 1992). A reduction in the C:F ratio will lead to a decrease in the abundance of *Prevotella_1*, thereby reducing the propionic acid content. Members of the genus *Succiniclasticum* can convert succinic acid into propionic acid, and, the content of *Succiniclasticum* in the cold season in this study was significantly lower than that in the warm season. In addition, the concentration of propionic acid in the cold season was significantly lower than that in the warm season (*P* < 0.05), which corresponds to the decrease in abundance of *Prevotella_1*. The reason for this phenomenon is the lack of nutrients in the cold season. The relative abundance of *Rikenellaceae_ RC9_gut_group* bacteria increased significantly in the cold season (*q* < 0.05) compared with the abundance in the warm season. Although the specific function of *Rikenellaceae RC9* is unknown, some studies have suggested that *Rikenellaceae RC9* bacteria are closely related to members of the genus *Alistipes* (Seshadri et al., 2018). *Rikenellaceae* may therefore play a role in degrading plant-derived polysaccharides like *Alistipes* (He et al., 2015; Peng et al., 2015). Based on this, it is speculated that the cellulose content of forage grasses increased in the cold season (the dry season) and the grazing Tibetan sheep exhibited an ultra-high degradation capacity for cellulosic polysaccharides, which allowed them to better adapt to cold season nutritional stress and the cold climate. Many cellulolytic bacterial genera showing seasonal variations were also identified in the rumen microbiota, such as *Ruminococcus_2*, *Fibrobacter*, *Butyrivibrio_2*, *Treponema_2*, and *Pseudobutyrivibrio* (Yang et al., 2010; Leng et al., 2011; Xue et al., 2016). Cellulolytic bacteria are an important class of bacteria that degrade cellulose in the rumen and consequently play a key role in the production of VFAs (Tajima et al., 2001; Abdul Rahman et al., 2016). Among these cellulolytic genera, *Ruminococcus_2* increased significantly in the cold season compared to the warm season (*P* < 0.05) and *Fibrobacter* also showed an increase in abundance in the cold season, but the difference was not significant (*P* > 0.05). This indicated that Tibetan sheep could degrade cellulose more effectively in the cold season, generating a large amount of VFAs. The genera *Butyrivibrio_2*, *Treponema_2*, and *Pseudobutyrivibrio* showed a significant decrease in relative abundance in the cold season compared to the warm season, which may be related to the production of low amounts of methane. Studies have reported that the number of *Vibrio butyrivibrio* (*Butyrivibrio*) is positively related to the number of methanogenic bacteria (Xue et al., 2016).

PICRUSt software was used to predict the gene function of the rumen microorganisms and revealed that there were significant differences in the gene function of microorganisms between the cold and warm seasons. KEGG gene family predictions showed that there were significant increases in functions related to energy metabolism in the cold season compared with the warm season. COG gene family predictions revealed that the function of energy production and conversion was also significantly increased in the cold season (*P* = 0.0002). Increases in these functions in Tibetan sheep may meet the energy metabolism needs of the sheep in the cold season. Genes with functions related to sugar biosynthesis and metabolism also showed a significant increase in the cold season (*P* = 0.0073). This was consistent with the results of Sun et al. (2016) and is conducive to the digestion of cellulose and hemicellulose. Therefore, the intestinal microbiota pattern found in Tibetan sheep in the cold season may improve the efficiency of food metabolism in the sheep during this season, helping them effectively deal with the harsh environment of the Plateau cold season.

The rumen epithelium is a unique location for host-microorganism interactions, and these interactions can affect the net utilization of nutrients throughout the host. Consequently, significant correlation exists between host genes and the microbiome (Lin et al., 2019). Therefore, expression of genes related to rumen epithelial nutrient absorption and barrier function were analyzed to obtain differential expression characteristics of rumen epithelium genes in cold and warm seasons. Studies have shown that nutrients are not only absorbed by the rumen epithelium in the form of VFAs, but some glucose is directly absorbed by *SGLT1* (Aschenbach et al., 2000a, 2002). In addition, studies have shown that the relative energy value ratio of ruminant glucose to VFAs in monogastric species is much higher because ruminants rely almost entirely on gluconeogenesis to meet their glucose needs (Reynolds et al., 1994). Direct absorption of glucose through *SGLT1* will reduce metabolic expenditure of gluconeogenesis in animals (Aschenbach et al., 2000b). Therefore, we speculate that expression of *SGLT1* in the rumen epithelium of Tibetan sheep during the cold season is a specific adaptive mechanism evolved in response to the lack of energy in the cold season. In this study, expression of *SGLT1* in the rumen epithelium in the cold season was significantly lower than that in the warm season (*P* < 0.05). This phenomenon may help prevent acidosis when the warm season pastures are adequate. Studies have shown that when the glucose concentration is increased in the rumen, *SGLT1* will be overexpressed in the rumen epithelial tissue to prevent or alleviate rumen acidosis (Aschenbach et al., 2002). Expression levels of rumen epithelial barrier genes *Claudin-4* and *ZO-1* are significantly lower in the cold season than in the warm season (*P* < 0.05). Some studies have shown that when the feed intake of ruminants decreases, permeability of the gastrointestinal tract barrier increases (Zhang et al., 2013a, b; Pederzolli et al., 2018). *Claudin-4* and *ZO-1*, as the main components of the rumen epithelial barrier, control the permeability of this barrier, thereby regulating the absorption of nutrients and preventing entry of harmful substances (Zhang et al., 2018; Aschenbach et al., 2019). In this study, the reduction in feed intake during the cold season resulted in decreased expression of barrier-related genes. In addition, a previous study found that a reduction in feed intake resulted in reduced absorption of VFAs and an increase in the permeability of the rumen epithelium to glucose (Gäbel et al., 2002). The results of our study were consistent with this.

**FIGURE 8:**
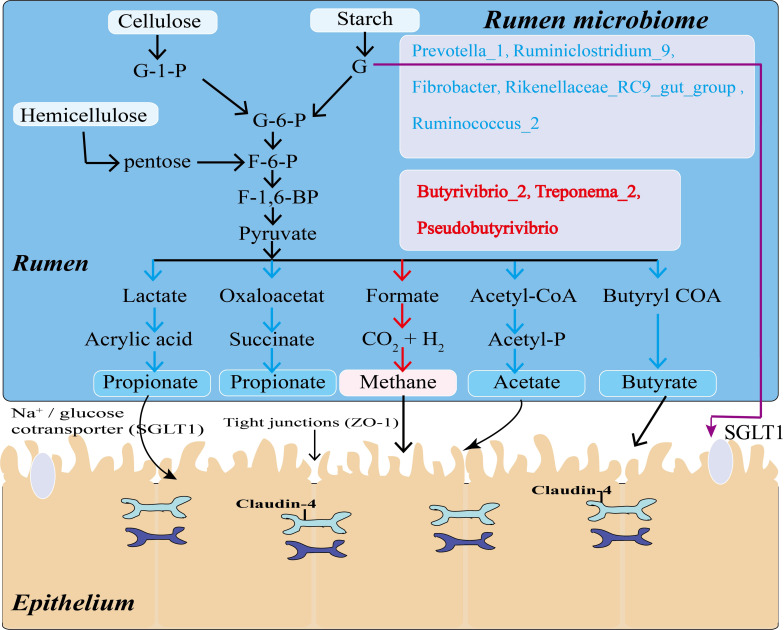
Model of mechanisms for generation and partial absorption of VFAs in Tibetan sheep. Microorganisms in blue and red lettering act on the pathways marked by blue and red arrows, respectively.

In summary, we evaluated the correlation between rumen microorganisms (genera-level), VFAs and rumen epithelial mRNA expression. A number of bacteria present in the rumen of Tibetan sheep significantly correlated with the rumen fermentation product acetate (*P* < 0.05) and with the rumen epithelial gene *SGLT1* and barrier-related genes (*Claudin-4*, *ZO-1*) (*P* < 0.05). Microorganisms in the rumen ferment chyme into VFAs, which are mainly absorbed by the rumen epithelial barrier, and some unfermented glucose is directly absorbed through the rumen epithelium into the blood by SGLT1 (Figure 8). This process indicates that there is a direct relationship between rumen microorganisms, VFAs, and host genes. Recent studies have identified specific correlation between microbe-host genes in the intestines of sheep and wild mice (Li et al., 2018; Suzuki et al., 2019), and there are similar findings in human studies (Ma et al., 2014; Blekhman et al., 2015). Our study used VFAs as intermediate products to analyze correlation between rumen microbes, rumen VFAs and host gene expression in the rumen epithelium. The identified correlation can explain the specific adaptability of Tibetan sheep to the cold season of the Qinghai-Tibet Plateau, and provides new insights for future research into the specific regulatory mechanisms of this adaptation.

## Data Availability Statement

The sequencing data were deposited into the Sequence Read Archive (SRA) of NCBI (Accession Nos. SRR12719079–SRR12719088).

## Ethics Statement

The animal study was reviewed and approved by Livestock Care Committee of Gansu Agricultural University (Approval No. GAU-LC-2020-27). Written informed consent was obtained from the owners for the participation of their animals in this study.

## Author Contributions

XL, JH, and YL designed the study. YS, RD, WZ, WL, HW, and HS performed the experiments and collected the samples. YS, JW, and SL analyzed the data. XL and YS wrote the manuscript. All authors contributed to the article and approved the submitted version.

## Conflict of Interest

The authors declare that the research was conducted in the absence of any commercial or financial relationships that could be construed as a potential conflict of interest.

## Supplementary Material

Supplementary Data Sheet 1 Differential species analysis; ANOVA analysis of variance.
