# Supplementary material for: Interactions Between Rumen Microbes, VFAs, and Host Genes Regulate Nutrient Absorption and Epithelial Barrier Function During Cold Season Nutritional Stress in Tibetan Sheep
Source: Front Microbiol. 2020 Nov 5;11:593062. doi: 10.3389/fmicb.2020.593062 (PMC7674685; doi:10.3389/fmicb.2020.593062)
Supplement: Supplementary file 2 [file Table_1.docx]

Table S1 Primer sequence information

| Gene | primer sequence（5'-3'） | length | temperature |
| --- | --- | --- | --- |
| *β-actin* | F:AGCCTTCCTTCCTGGGCATGGA | 113bp | 60℃ |
|  | R:GGACAGCACCGTGTTGGCGTAGA |  |  |
| *SGLT1* | F:GTGCAGTCAGCACAAAGTGG | 198bp | 60℃ |
|  | R:CCCGGTTCCATAGGCAAACT |  |  |
| *Claudin-4* | F:AAGGTGTACGACTCGCTGCT | 237bp | 60℃ |
|  | R:GACGTTGTTAGCCGTCCAG |  |  |
| *ZO-1* | F:CGACCAGATCCTCAGGGTAA | 161bp | 60℃ |
|  | R:AATCACCCACATCGGATTCT |  |  |
