# Supplementary material for: Interactions Between Rumen Microbes, VFAs, and Host Genes Regulate Nutrient Absorption and Epithelial Barrier Function During Cold Season Nutritional Stress in Tibetan Sheep
Source: Front Microbiol. 2020 Nov 5;11:593062. doi: 10.3389/fmicb.2020.593062 (PMC7674685; doi:10.3389/fmicb.2020.593062)
Supplement: Supplementary file 3 [file Table_2.docx]

| Sample ID | PE Reads | Raw Tags | Clean Tags | Effective Tags | AvgLen(bp) | GC(%) | Q20(%) | Q30(%) | Effective(%) |
| --- | --- | --- | --- | --- | --- | --- | --- | --- | --- |
| A1 | 79998 | 77142 | 73929 | 69291 | 419 | 54.72 | 97.74 | 95.68 | 86.62 |
| A2 | 80064 | 77374 | 74285 | 69789 | 419 | 54.58 | 97.81 | 95.8 | 87.17 |
| A3 | 79767 | 77330 | 74073 | 69275 | 419 | 54.53 | 97.87 | 95.89 | 86.85 |
| A4 | 79945 | 77132 | 73683 | 67048 | 418 | 54.87 | 97.79 | 95.75 | 83.87 |
| A5 | 79789 | 77428 | 74609 | 70381 | 418 | 54.2 | 97.87 | 95.9 | 88.21 |
| B1 | 79901 | 77448 | 74290 | 72538 | 419 | 53.73 | 97.77 | 95.74 | 90.78 |
| B2 | 79696 | 77397 | 74541 | 72957 | 418 | 53.88 | 97.81 | 95.82 | 91.54 |
| B3 | 79829 | 77601 | 74826 | 73077 | 419 | 53.76 | 97.87 | 95.92 | 91.54 |
| B4 | 79978 | 77344 | 73998 | 72608 | 419 | 53.99 | 97.77 | 95.74 | 90.78 |
| B5 | 79865 | 77218 | 73992 | 72636 | 419 | 53.66 | 97.84 | 95.88 | 90.95 |

Table S2 Sample sequencing data statistics
