# Supplementary material for: Interactions Between Rumen Microbes, VFAs, and Host Genes Regulate Nutrient Absorption and Epithelial Barrier Function During Cold Season Nutritional Stress in Tibetan Sheep
Source: Front Microbiol. 2020 Nov 5;11:593062. doi: 10.3389/fmicb.2020.593062 (PMC7674685; doi:10.3389/fmicb.2020.593062)
Supplement: Supplementary file 4 [file Image_1.pdf]

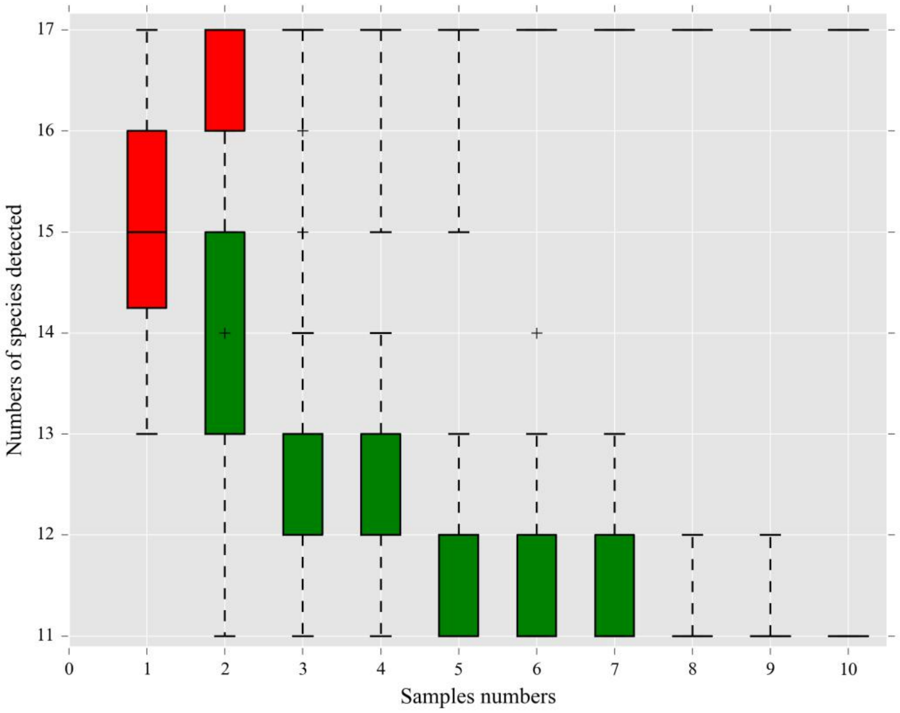

A

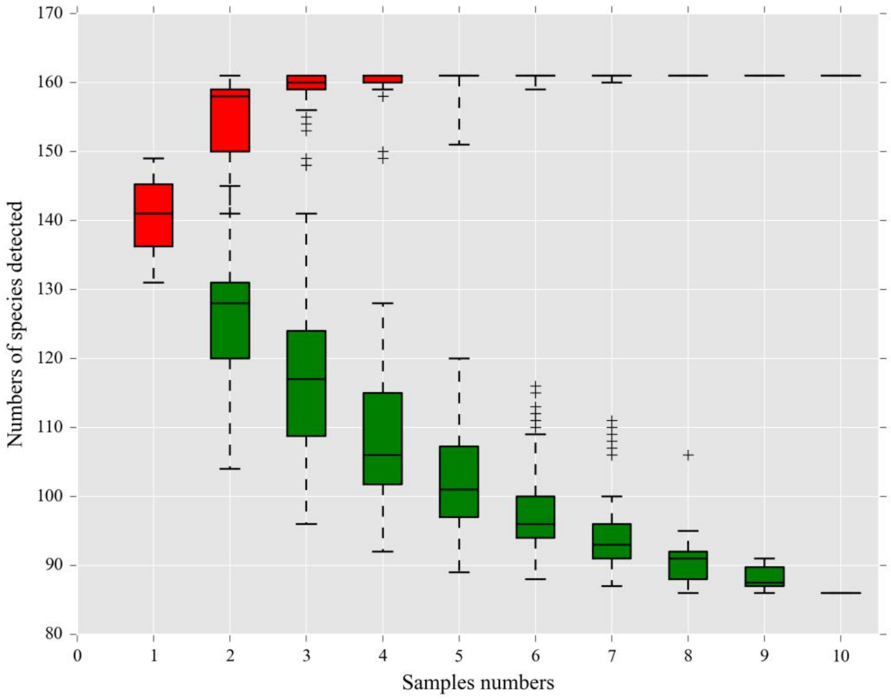

B

Figure. S1 Species accumulation curve (A: phylum level; B: genus Level)

Note: The abscissa represents the sample size; Ordinate represents the number of species after

sampling; The red box line constitutes the accumulation curve; The green box line consists of a common quantity curve
