# Supplementary figures and images for: Interactions Between Rumen Microbes, VFAs, and Host Genes Regulate Nutrient Absorption and Epithelial Barrier Function During Cold Season Nutritional Stress in Tibetan Sheep
Source: Front Microbiol. 2020 Nov 5;11:593062. doi: 10.3389/fmicb.2020.593062 (PMC7674685; doi:10.3389/fmicb.2020.593062)

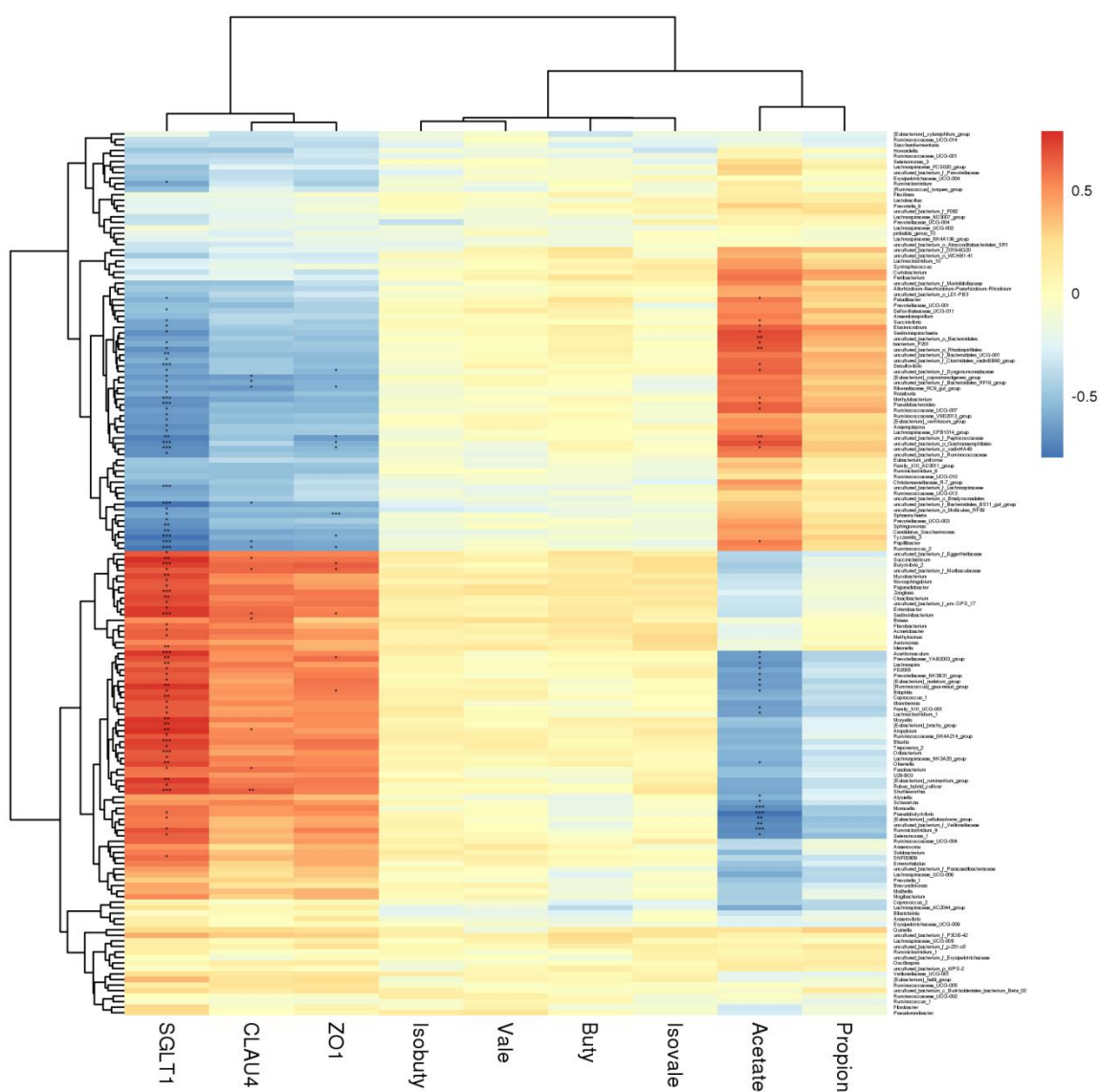

Figure. S2 Heat map of correlation between rumen microbe - Rumen VFAs-mRNA expression

Supplement: Supplementary file 5 [file Image_2.pdf]
